# Antibiotic use and risk of non-Hodgkin's lymphoma: a population-based case–control study

**DOI:** 10.1038/sj.bjc.6604127

**Published:** 2007-12-04

**Authors:** L A Anderson, G Gridley, E A Engels, L M Morton, J R Cerhan, W Cozen, R K Severson, S Davis, P Hartge, M S Linet

**Affiliations:** 1 Division of Cancer Epidemiology and Genetics, National Cancer Institute, Bethesda, MD 20892, USA; 2 Office of Preventative Oncology, Division of Cancer Prevention, National Cancer Institute, Bethesda, MD 20892, USA; 3 Department of Health Sciences Research, Mayo Clinic College of Medicine, Rochester, MN 55905, USA; 4 The University of Iowa, Iowa City, IA 52242, USA; 5 Department of Preventive Medicine, University of Southern California, Los Angeles, CA 90033, USA; 6 Department of Pathology, University of Southern California, Los Angeles, CA 90033, USA; 7 Karmanos Cancer Institute, Detroit, MI 48201, USA; 8 Department of Family Medicine and Public Health Sciences, Wayne State University, Detroit, MI 48202, USA; 9 Fred Hutchinson Cancer Research Center, Seattle, WA 19024, USA; 10 School of Public Health and Community Medicine, University of Washington, Seattle, WA 98195, USA

**Keywords:** lymphoma, non-Hodgkin, antibiotics, case–control study, epidemiology

## Abstract

Antibiotic use in 759 non-Hodgkin's lymphoma (NHL) patients and 589 controls was compared. Neither total antibiotic use (odds ratio=0.7, 95% confidence interval=0.5–1.2), nor antibiotic use by site, was associated with total NHL, or NHL subtypes. There were no trends with frequency or age at first use (*P* trend=0.23 and 0.26, respectively).

The human immunodeficiency virus (HIV) epidemic during the 1980s was paralleled by a 3–4% annual increase in the incidence of non-Hodgkin's lymphoma (NHL) (Hartge *et al*, 1994). However, NHL incidence had been increasing since before the 1950s (Zheng *et al*, 1992), with some levelling off in recent years (Eltom *et al*, 2002; Morton *et al*, 2006). The dramatic rise in antibiotic use since the 1940s has led some scientists to hypothesise that antibiotics may increase the risk of developing NHL. *In vitro* evidence suggests that certain antibiotics are cytotoxic (Robbana-Barnat *et al*, 1997; Summan and Cribb, 2002) or genotoxic (Snyder and Green, 2001), and some may have immunomodulatory effects (Van *et al*, 1996). Moreover, chronic infections treated with antibiotics, such as tuberculosis, have been associated with NHL (Swerdlow, 2003). While case–control studies of self-reported antibiotic use have found a 30–90% increased risk of NHL (Bernstein and Ross, 1992; Kato *et al*, 2003; Chang *et al*, 2005), no associations were observed in case–control studies utilising pharmacy (Beiderbeck *et al*, 2003) or medical record data (Doody *et al*, 1996). We investigated the association between NHL and antibiotics in a large multicentre case–control study (1998–2000) within the United States.

## MATERIALS AND METHODS

Study methods have been described in detail elsewhere (Chatterjee *et al*, 2004; Engels *et al*, 2004; Hartge *et al*, 2005). Briefly, 1322 newly diagnosed NHL cases were identified from four geographical regions covered by the National Cancer Institute's population-based Surveillance, Epidemiology, and End Results (SEER) cancer registries. All cases residing in Iowa and Seattle, and all African-American cases and a random sample of Caucasian cases residing in Detroit and Los Angeles, aged 20–74 years, with a first primary diagnosis of NHL were eligible. Cases were histologically confirmed and coded according to the International Classification of Diseases-Oncology, 2nd Edition (Percy *et al*, 1990). NHL cases with known HIV infection were excluded.

Eligible controls were aged 20–74 years, resided in one of the four geographical regions (Iowa, Seattle, Detroit, or Los Angeles) and had no known history of HIV infection or NHL. Controls younger than age 65 were identified using a one-step random digit dialing approach (Casady and Lepowski, 1993). Controls aged 65 years or older were identified from Medicare eligibility files. Controls were frequency matched to the distribution of cases by age (5-year age groups), race, gender, and geographic area.

Human subject review boards at the National Cancer Institute and all participating institutions approved the study. All subjects provided written informed consent.

To accommodate a large number of questions the study population was divided into two groups, each receiving a different version of the computer-assisted personal interview (CAPI). Our analyses are focused on the group, including all African-American and one half of all other participants chosen at random, who completed the CAPI consisting of a detailed medical history questionnaire.

Subjects were asked to recall if they had ever taken antibiotics, in the past 1 year, for infections by organ site and procedure. Information was also obtained about antibiotics used ⩾5 days for conditions/procedures not listed in the questionnaire. These data were combined to create an indicator of having ever used antibiotics. For each positive response information was gathered about the number of times antibiotics were used and the age at first use.

Of the 1332 NHL patients assigned to receive the medical history version of the CAPI, 1017 (76.4%) were contacted. Of these, 175 refused to participate and 83 did not participate for other reasons including serious illness, cognitive impairment or failure to respond to multiple contacts. In total, 759 NHL cases (74.6% of cases approached, 57.0% of eligible cases) participated.

Of the 1419 controls randomly assigned to complete the medical history CAPI, 1196 (84.3%) were contacted. Of these, 525 refused and 82 did not participate for other reasons including serious illness, cognitive impairment, and failure to respond to multiple contacts. Therefore, 589 controls (49.2% of potential controls approached, 41.5% of eligible controls) participated.

Unconditional logistic regression analyses in STATA 10.0 (Stata Corp., College Station, TX, USA) were used to estimate odds ratios (ORs) and 95% confidence intervals (CIs) for the associations between NHL and history of antibiotic use, frequency of use and age at first use of antibiotics (in tertiles). Tertile cut points were based on the distributions among the controls. Tests for linear trend across categories were calculated; reported *P*-values are two-sided. Additional analyses were performed for the two most common NHL subtypes, diffuse large B-cell lymphoma (DLBCL) and follicular lymphoma, using polytomous logistic regression. Analyses were adjusted for age, gender, race, study site, and education.

## RESULTS

Cases were similar to controls with respect to age (*P*=0.68), gender (*P*=0.38), and education (*P*=0.74) but were less likely to be African-American (*P*=0.03) (Table 1).

Overall, antibiotic use was not associated with an increased risk of NHL or the DLBCL or follicular subtypes. In addition, no significant trends were observed with increasing frequency, or increasing age at first use, of antibiotics, Table 2. No significant associations were observed when we stratified the analyses by age, centre, or education (data not shown).

More specifically, there were no significant associations between NHL overall, or NHL subtypes, and use of antibiotics to treat infections of the upper respiratory tract, eye, ear, gallbladder, urinary tract, bowel, brain, prostate, or skin, or antibiotics used to treat pneumonia, tuberculosis or acne, or antibiotics used before or after dental surgery (data not shown). In addition, no significant trends were observed between NHL and increasing frequency of use, or age at first use, of antibiotics for these conditions (data not shown). However, antibiotic use for genital infections was inversely associated with NHL (OR=0.59, 95% CI=0.39–0.90).

## DISCUSSION

Using data collected as part of a large, population-based, case–control study, we found that antibiotic use was not associated with an increased risk of NHL overall, or of the two major subtypes, DLBCL, or follicular lymphoma (FL). An inverse association between antibiotics used to treat genital infections and NHL was observed.

A nested case–control study within the PHARMO database of drug dispensing records in the Netherlands also reported no significant association between ever use of antibiotics and NHL (OR=0.81, 95% CI=0.51–1.26) (Beiderbeck *et al*, 2003). Another study, from two Kaiser Permanente medical care programmes, found no association between use of chloramphenicol and NHL, but this study lacked information on other antibiotics (Doody *et al*, 1996).

In contrast, most interview-based case–control studies have reported that antibiotics are associated with a 30–40% increased risk of NHL (Bernstein and Ross, 1992; Chang *et al*, 2005) and Kato *et al* (2003), found systemic use of penicillin to be associated with NHL (OR=1.6, 95% CI=1.1–2.2). These investigators have postulated that their findings could be due to a direct carcinogenic effect of antibiotics, differential recall bias or the possibility that frequent antibiotic use could be a surrogate marker for chronic infections or a compromised immune system (Kato *et al*, 2003; Chang *et al*, 2005).

Recall bias is a concern in case–control studies. Poor correlation between self-reported medication use and prescription records has been reported (van den Brandt *et al*, 1991; West *et al*, 1995). In an attempt to improve recall of self-reported antibiotic usage, we used a method, described by Klungel *et al* (2000) in which participants are asked to recall their use of medications for a range of conditions. A similar methodological approach was used in the case–control study by Kato *et al* (2003); however, our study was larger and used a more comprehensive assessment of antibiotic use. In addition, subjects were asked to recall medication usage more than 1 year prior to diagnosis to reduce the possibility of reverse causality. Information was not collected about antibiotic type as it would be difficult to elucidate lifetime use of specific antibiotics from a case–control study; thus, we were unable to determine if specific antibiotic types were associated with NHL. Using diagnostic information from four SEER cancer registries, stratified analyses showed no significant associations between antibiotic use and the two main subtypes of NHL: DLBCL and follicular lymphoma. Although our participation rates for controls were not optimal and could potentially introduce bias, stratification by factors that differed between participants and non-participants (including age, centre, and education) resulted in no significant associations with NHL, suggesting that major biases are unlikely.

An inverse association was observed between antibiotic use for genital infections and NHL. However, the lack of a biologically plausible explanation for this association suggests that it may have resulted by chance since multiple testing was not controlled for in the stratified analyses.

In summary, our results suggest that antibiotic use does not increase NHL risk. Antibiotics are a diverse group of drugs with different mechanisms of action and biological effects (Van *et al*, 1996). Difficulty of assessing use of specific antibiotics in questionnaire-based case–control studies, as well as lack of biological plausibility for a causative association between non-specific antibiotic use and NHL, suggests that future assessments of this hypothesis should be conducted using cohort studies, with documentation of specific antibiotic use from medical records, or large pharmaceutical data sets.

## Figures and Tables

**Table 1:** Descriptive characteristics of NHL cases and controls

**Variable**	**Controls, no. (%^a^)**	**Cases, no. (%^a^)**
*Age(years)*		
<30	17 (3)	19 (3)
30–39	54 (9)	63 (8)
40–49	86 (15)	133 (18)
50–59	139 (24)	183 (24)
60–69	188 (32)	240 (32)
70+	105 (18)	121 (16)
		
*Gender*		
Male	304 (52)	410 (54)
Female	285 (48)	349 (46)
		
*Race*		
African-American	145 (25)	100 (13)
White	407 (69)	604 (80)
Others	37 (6)	55 (7)
		
*Education*		
<12 years	61 (10)	85 (11)
12–15 years	361 (61)	471 (62)
>15 years	167 (28)	202 (27)
		
*Study centre*		
Detroit	138 (23)	238 (32)
Iowa	124 (21)	170 (22)
Los Angeles	172 (29)	187 (25)
Seattle	155 (26)	164 (22)
		
*NHL subtype*		
Diffuse large B-cell lymphoma		224 (30)
Follicular lymphoma		172 (23)
T-cell lymphoma		54 (7)
Other/unclassifiable		309 (41)

a Percentages may not sum to 100 because of rounding.

**Table 2:** Adjusted^a^ ORs and 95% CI for NHL (all, diffuse large-B-cell, and follicular) for use of antibiotics

		**All NHL cases**			**DLBCL**			**Follicular lymphoma**		
	**Controls**	**Cases**	**OR**	**95% CI**	**Cases**	**OR**	**95% CI**	**Cases**	**OR**	**95% CI**
*Any antibiotic use*										
Never	35	53	1	Reference	11	1	Reference	11	1	Reference
Ever	528	673	0.72	0.45–1.16	204	1.03	0.49–2.18	154	0.77	0.36–1.63
										
*Frequency of antibiotic use*										
0–1 times	84	109	1	Reference	25	1	Reference	22	1	Reference
2–4 times	96	114	0.87	0.58–1.31	38	1.27	0.69–2.35	20	0.81	0.41–1.63
4–19 times	209	289	0.95	0.67–1.36	84	1.13	0.65–1.97	74	1.13	0.64–2.00
⩾20 times	177	217	0.78	0.53–1.14	69	1.00	0.56–1.80	49	0.78	0.42–1.44
			*P trend*=0.26			*P trend*=0.74			*P trend*=0.57	
*Age at first use of antibiotics*										
Never	35	53	1	Reference	11	1	Reference	11	1	Reference
<15	171	248	0.78	0.47–1.31	88	1.21	0.56–2.69	62	0.90	0.40–2.00
15–25	142	167	0.69	0.42–1.15	51	1.04	0.47–2.31	26	0.52	0.22–1.19
>25	209	252	0.73	0.45–1.20	61	0.92	0.42–2.01	64	0.91	0.42–1.98
			*P trend*=0.28			*P trend*=0.32			*P trend*=0.86	

CI=confidence interval; ORs=odds ratio.

a Adjusted for age (in decades), gender, race (white, non-white), study site (Iowa, Seattle, Detroit, Los Angeles), and level of education (<12, 12–15, and >15 years).
